# High-throughput phenotyping allows the selection of soybean genotypes for earliness and high grain yield

**DOI:** 10.1186/s13007-022-00848-4

**Published:** 2022-02-02

**Authors:** Dthenifer Cordeiro Santana, Marcos Paulo de Oliveira Cunha, Regimar Garcia dos Santos, Mayara Fávero Cotrim, Larissa Pereira Ribeiro Teodoro, Carlos Antonio da Silva Junior, Fabio Henrique Rojo Baio, Paulo Eduardo Teodoro

**Affiliations:** 1 Universidade Estadual Paulista “Júlio de Mesquita Filho” (UNESP), Campus de Ilha Solteira, Ilha Solteira, SP 15385-000 Brazil; 2 Universidade Federal de Mato Grosso do Sul (UFMS), Campus de Chapadão do Sul, Chapadão do Sul, MS 79560-000 Brazil; 3 Department of Geography, Universidade do Estado do Mato Grosso (UNEMAT), Campus de Sinop, Sinop, MT 78555-000 Brazil

**Keywords:** *Glycine max* (L). Merrill, Plant breeding, Precision agriculture, Spectral models, Vegetation indices

## Abstract

**Background:**

Precision agriculture techniques are widely used to optimize fertilizer and soil applications. Furthermore, these techniques could also be combined with new statistical tools to assist in phenotyping in breeding programs. In this study, the research hypothesis was that soybean cultivars show phenotypic differences concerning wavelength and vegetation index measurements.

**Results:**

In this research, we associate variables obtained via high-throughput phenotyping with the grain yield and cycle of soybean genotypes. The experiment was carried out during the 2018/2019 and 2019/2020 crop seasons, under a randomized block design with four replications. The evaluated soybean genotypes included 7067, 7110, 7739, 8372, Bonus, Desafio, Maracai, Foco, Pop, and Soyouro. The phenotypic traits evaluated were: first pod height (FPH), plant height (PH), number of branches (NB), stem diameter (SD), days to maturity (DM), and grain yield (YIE). The spectral variables evaluated were wavelengths and vegetation indices (NDVI, SAVI, GNDVI, NDRE, SCCCI, EVI, and MSAVI). The genotypes Maracai and Foco showed the highest grain yields throughout the crop seasons, in addition to belonging to the groups with the highest means for all VIs. YIE was positively correlated with the NDVI and certain wavelengths (735 and 790 nm), indicating that genotypes with higher values for these spectral variables are more productive. By path analyses, GNDVI and NDRE had the highest direct effects on the dependent variable DM, while NDVI had a higher direct effect on YIE.

**Conclusions:**

Our findings revealed that early and productive genotypes can be selected based on vegetation indices and wavelengths. Soybean genotypes with a high grain yield have higher means for NDVI and certain wavelengths (735 and 790 nm). Early genotypes have higher means for NDRE and GNDVI. These results reinforce the importance of high-throughput phenotyping as an essential tool in soybean breeding programs.

## Background

Soybean (*Glycine Max* (L.) Merrill) is the most important oilseed crop and a major commodity worldwide. The crop complex in Brazil [a combination of grains and main derivatives (oil and bran)] surpassed that of the USA in the 2019/2020 crop season [1]. Due to large-scale world population growth combined with unstable product prices, the demand for quality raw materials and fair prices has increased [2], requiring highly productive cultivars and increasingly efficient farming systems.

In this sense, Brazilian soybean breeding programs have sought cultivars that combine high grain yield and earliness. Earliness has been a target because it allows farmers to grow corn or cotton in the off-season, after soybean cultivation (in-season). Furthermore, early-cycle genotypes remain less time in the field and are subject to less disease pressure [3]. However, plant cycle characterization is a time and labor-demanding task since it requires on-field counting of the number of days from emergence until flowering or maturation of each genotype. This is because hundreds of soybean genotypes are evaluated annually in the breeding programs, and the cycle monitoring for each plot must be performed daily. To overcome these difficulties, the use of remote sensing techniques emerges as a high potential tool, providing specific and large-scale information for crop assessment [4–7].

Remote sensing-based high-throughput phenotyping (HTP) is a reliable and fast approach to real-time and large-scale plant trait measurements [8–10]. Adopting this approach is essential to achieve greater efficiency of plant breeding, as it provides monitoring and decision support with applications in several scenarios, such as monitoring the plant status [8, 11–13], discriminating cultivars [4, 6], predicting crop yield [7, 14, 15], and selecting genotypes for traits of interest [9, 16, 17]. Unmanned Aerial Vehicles (UAVs) are essential for remote sensing-based HTP since they provide fast real-time data via remote sensors. These tools are required for obtaining Vegetation Indices (VIs), which are mathematical models for different wavelengths [7, 10, 18]. UAVs can estimate the spectral component of vegetation through combinations between red and near-infrared spectral bands [19] and can be assembled to assess the growth vigor, nutrient status, and photosynthetic activity of the plants in the field [20–22]. Thus, the use of spectral variables obtained by UAV imaging shows to be a promising approach for reliable, faster, and cost-effective measurements of the cycle and yield-related traits in soybean.

Santana et al. [11] assessed the relationship between VIs obtained from UAV multispectral imagery and leaf N content and yield-related traits in corn varieties grown in different N topdressing levels, and they verified a positive relationship between NDVI and NDRE and grain yield under adequate N levels. Da Silva et al. [7], in a study aiming at identifying which VIs can be used in soybean grain yield prediction by using UAV and remote multispectral sensor, verified that NDVI and SAVI had the higher direct effect on grain yield. However, further studies assessing the relationship between VIs and cycle and yield-related traits in soybean cultivars are still needed. Identifying the cause-and-effect relationship between spectral and agronomic variables provides an easier and faster phenotyping process in breeding programs since efforts can be directed only to the wavelengths and VIs showing the highest cause-and-effect relationship with cycle and yield. Additionally, genotypes with better means for these spectral variables should be identified to achieve an efficient selection for yield and earliness.

The research hypothesis was that soybean cultivars show phenotypic differences concerning the measurements of wavelengths and VIs. Thus, the objective of this study was to identify variables obtained by UAV-based HTP that are related to the grain yield and cycle of soybean genotypes.

## Methods

### Field trials

During the 2018/2019 and 2019/2020 crop seasons, two experiments were carried out at the experimental field of the Federal University of Mato Grosso do Sul, campus of Chapadão do Sul (18° 46′ 26″ S, 52° 37′ 28″ W, mean altitude of 810 m). According to the Köppen classification system, the climate is classified as tropical savanna (Aw). The soil of the experimental area is classified as dystrophic Red Latosol with a clay texture [23], and has the following chemical characteristics in the 0–20 cm layer, according to International System of Units: pH (CaCl_2_) = 5.0; H + Al = 35.0 mmol_c_ dm^−3^; Ca = 26.0 mmol_c_ dm^−3^; Mg = 6.0 mmol_c_ dm^−3^; K = 55.0 mg dm^−3^; P = 16.0 mg dm^−3^; S = 24.0 mg dm^−3^; B = 0.46 mg dm^−3^; Cu = 0.7 mg dm^−3^; Fe = 25.0 mg dm^−3^; Mn = 10.4 mg dm^−3^; Zn = 5.2 mg dm^−3^; OM = 24.0 g dm^−3^; CEC = 68.0 mmol_c_ dm^−3^; and base saturation = 48.8%.

**Fig. 1:**
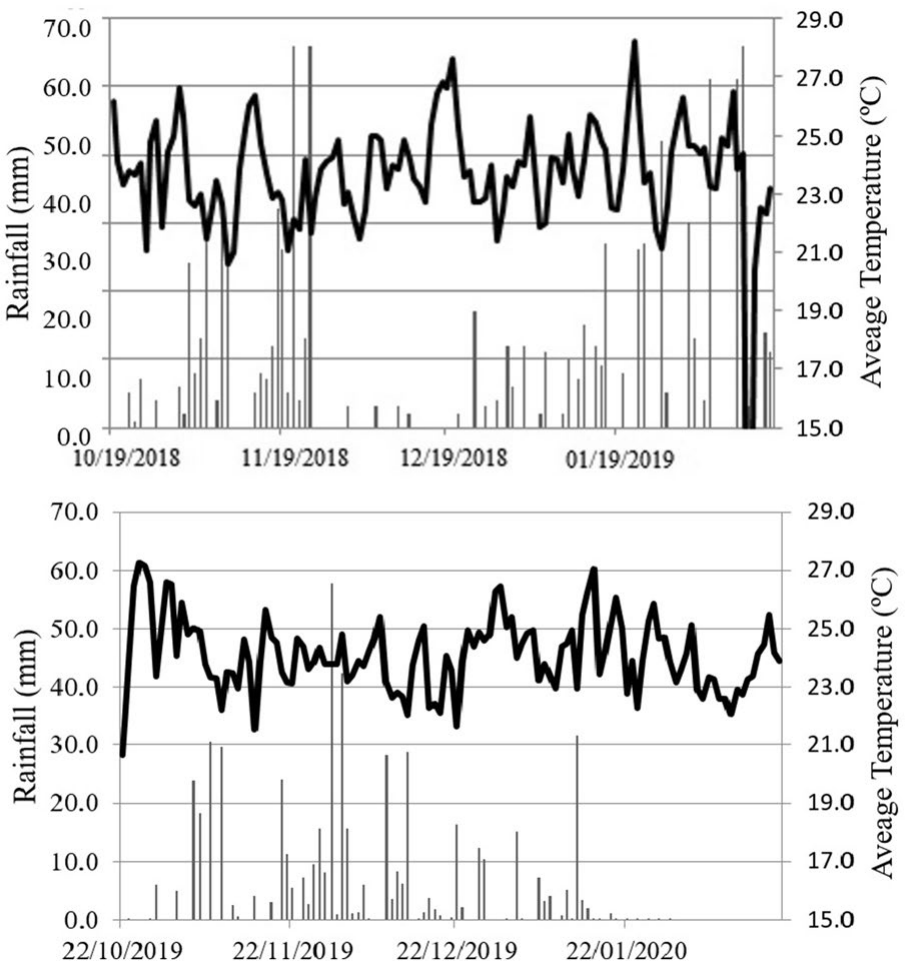
Weather conditions during the 2018/2019 and 2019/2020 crop seasons

The experimental design consisted of randomized blocks with four replications. The evaluated soybean genotypes included 7067, 7110, 7739, 8372, Bonus, Desafio, Maracai, Foco, Pop, and Soyouro. The main phenotypic traits of the cultivars are shown in Table 1. The plots consisted of five rows (4 m long) with a spacing of 0.45 m. The sowing density was 15 plants m^−1^. The climatic conditions during the experiments are shown in Fig. 1, respectively.

**Table 1:** Characterization of evaluated soybean cultivars

Genotypes	RMG	Growth habit	Other observations
7067	6.7	Semideterminate	Resistant to asian rust (*Phakopsora pachyrhizi*), stem canker (*Diaporthe phaseolorum*) and bacterial pustule (*Xanthomonas axonopodis* pv. *glycines*)
7110	6.8	Indeterminate	Resistant to bacterial pustule (*Xanthomonas axonopodis* pv. *glycines*), frog eye leaf spot (*Cercospora sojina*), macrophomina (*Macrophomina phaseolina*)
7739	7.7	Semideterminate	Resistant to lodging and races 1 and 3 of the soybean cyst nematode (*Heterodera glycines*)
8372	8.3	Determinate	Resistant to races 1 and 3 of the soybean cyst nematode (*Heterodera glycines*) and bacterial pustule (*Xanthomonas axonopodis* pv. *glycines*)
Bonus	7.9	Indeterminate	Resistant to stem canker (*Diaporthe phaseolorum*)
Desafio	7.4	Indeterminate	Resistant to lodging and stem canker (*Diaporthe phaseolorum*)
Maracai	7.7	Indeterminate	Resistant to races 3, 6, 9, 10 and 14 of the soybean cyst nematode (*Heterodera glycines*) and stem canker (*Diaporthe phaseolorum*)
Foco	7.2	Indeterminate	Resistant to races 3 and 14 of the soybean cyst nematode (*Heterodera glycines*) and stem canker (*Diaporthe phaseolorum*)
Pop	6.4	Indeterminate	Resistant to stem canker (*Diaporthe phaseolorum*)
Soyouro	7.1	Indeterminate	Resistant to lodging and stem canker (*Diaporthe phaseolorum*)

*RGM* relative maturity group

### Evaluated variables

During both crop seasons, the high-throughput phenotyping measurements were performed at 60 days after emergence (DAE). On this date, soybean cultivars were in R1 and R2 according to phenological scale of Fehr and Caviness [24], showing the highest vegetative peak and maximum physiological development [25]. For spectral data acquisition, we used a Sensefly eBee RTK fixed-wing unmanned aerial vehicle (UAV), with autonomous take-off control, flight plan, and landing. The overflights were performed with 75% lateral and 80% longitudinal overlap of the taken images. The overflight was performed at 100 m altitude, allowing a spatial image resolution of 0.10 m. The aircraft can fly between 50 and 400 ha field per flight (at a fully charged battery and dependent of the needed spatial resolution, wind speed and flight altitude). The flight autonomy depends on the image’s desired spatial resolution and the overlap of the passes or flight lines, which can be up to 45 min. The aircraft has 0.96 m of wingspan, and the weight without a camera and battery is 0.46 kg. The nominal take-off weight with camera e battery is 0.73 kg. The nominal cruise speed is between 40 and 90 km h^−1^ (wind dependent).

SenseFly eBee RTK was equipped with a luminosity sensor and the Parrot Sequoia multispectral camera, with 1280 × 960 pixels and pixel size of 3.75 × 3.75 µm (Focal Length of 3.98 mm). The Parrot Sequoia includes a sunshine sensor at the top of the equipment, which registers the sun’s total spectral irradiance at-sensor level and, thus, facilitates the automatic determination of the at-sensor reflectance. The assumed Full-Width Half Maximum (FWHM) provided in the specification sheet, by guessing the shape of the relative spectral response function [26], are: Green 530–570 nm; Red 640–680 nm; Red-edge 730–740 nm; and NIR 770–810 nm. The overflights were carried out near the zenith due to the minimization of the shadows of the trees, at 11 a.m., given that the multispectral sensor is passive type, that is, dependent on the solar luminosity.

The following wavelengths were evaluated: green (550 nm), red (660 nm), near-infrared (735 nm), and infrared (790 nm). The information acquired in these wavelengths allowed calculating the different vegetation indices, as shown in Table 2. The aerial survey was carried out using Real-Time Kinematics (RTK) technology, which was used to estimate the position of the camera at the time of image collection, with an accuracy of 2.5 cm. The images were mosaiced and orthorectified using the Pix4Dmapper software package. The positional accuracy of the orthoimages was verified using ground control points (GCP), obtained via data surveys in combination with RTK. A calibration reference plaque (calibration target) is also used, in the Pix4DMapper software, to convert the digital number of the pixels into reflectance values.

**Table 2:** Vegetation index (VIs) equations generated from high-throughput phenotyping and its respective reference

VIs	Equation	References
NDVI	$\frac{Nir - Red}{{Nir + Red}}$	[43]
SAVI	$\left( {1 + 0.5} \right)\frac{nir - red}{{nir + red + 0.5}}$	[44]
GNVDI	$\frac{Nir - Green}{{Nir + Green}}$	[45]
NDRE	$\frac{nir - rededge}{{nir + rededge}}$	[37]
SCCCI	$\frac{NDRE}{{NDVI}}$	[46]
EVI	$\frac{nir - red}{{\left( {nir + 6red - 7.5green} \right) + 1}}$	[47]
MSAVI	$\frac{{2Nir + 1 - \sqrt {\left( {2Nir + 1} \right)^{2} } - \left( {8Nir - Red} \right)}}{2}$	[48]

**Fig. 2:**
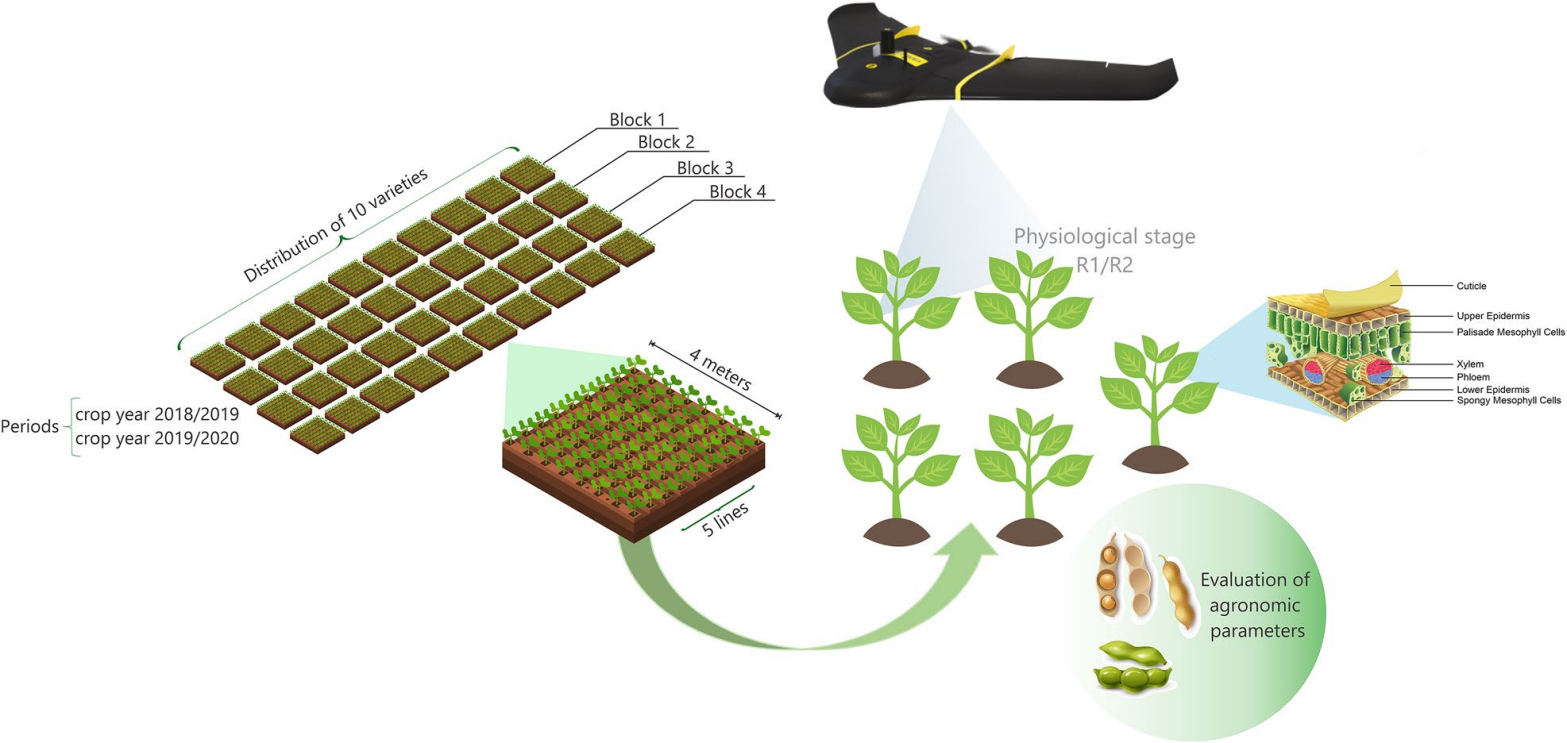
Diagram of ground data collection

From each plot, five plants were randomly selected to evaluate the following agronomic traits: first pod height (FPH, cm), plant height (PH, cm), main stem diameter (SD, cm), hundred grain mass (HGM), days to maturity (DM), and grain yield (YIE). A measuring tape was used to evaluate both the FPH and PH. The SD was assessed with the aid of a digital caliper. The DM corresponded to the number of days between the emergence and maturity of the plants. The HGM was assessed using an analytical precision balance and corrected to 13% humidity. The central row of each plot was manually harvested to evaluate the YIE, which was then corrected for 13% humidity and extrapolated to kg ha^−1^. Figure 2 demonstrates a diagram of ground data collection.

### Statistical analysis

The data were submitted to individual analyses of variance, considering all effects as fixed. After verifying that the ratio between the largest and smallest mean squared errors did not exceed 7.0, a joint analysis was performed in accordance with the model described in Eq. 1. The Scott–Knott test [27] was used for grouping the means.

1 $$ Y_{ijk} = \mu + B_{k} + G + S_{j} + G \times S_{ij} + \varepsilon_{ijk} , $$

where $Y_{ijk}$ is the observation for the k-th block evaluated in the i-th cultivar during the j-th crop season; $B_{k}$ is the fixed block effect; $G_{i}$ is the fixed genotype effect; $S_{j}$ is the random crop season effect; $G \times S_{ij}$ is the random interaction between genotypes and crops; and $\varepsilon_{ijk}$ is the error associated with observation $Y_{ijk}$.

Pearson’s correlations (r) between the evaluated trait pairs were estimated according to Eq. 2:

2 $$ r = \frac{{COV_{{\left( {xy} \right)}} }}{{\sqrt {\hat{\sigma }_{x}^{2} } \times \hat{\sigma }_{y}^{2} }}, $$

where *COV*_*(XY)*_ is the covariance between traits X and Y; $\hat{\sigma }_{x}^{2}$ is the variance of trait X; and $\hat{\sigma }_{y}^{2}$ is the variance of the YIE.

The graphical expression was performed using the functional relationship between the correlation coefficient estimates of the different environments, using a correlation network generated using Rbio software [28], in which the proximity between the nodes (traces) was proportional to the total value of the correlation between these nodes [29]. The thickness of the edges was controlled by applying a 0.60 cut-off value, in which only |rij| ≥ 0.60 have their edges highlighted. Thus, positive correlations were highlighted in green, while negatives correlations were highlighted in red.

The path analysis, considering YIE or DM as the principal dependent variable and the wavelengths and VIs as explanatory variables, was conducted according to the model described in Eqs. 3 and 4:

3 $$ YIE = \hat{\beta }_{1} 550 + \hat{\beta }_{2} 660 + \cdots + \hat{\beta }_{11} MSAVI + p_{\varepsilon } , $$

4 $$ DM = \hat{\beta }_{1} 550 + \hat{\beta }_{2} 660 + \cdots + \hat{\beta }_{11} MSAVI + p_{\varepsilon } , $$

where $\upbeta _{1} , \,\upbeta _{2} , \cdots\upbeta _{11}$ are the direct effects for the variables 550, 660, 735, 790, NDVI, SAVI, GNVDI, NDRE, SCCCI, EVI, and MSAVI; and ${\text{p}}_{\upvarepsilon }$ is the residual effect. All statistical analyses were performed using Genes [30], Sisvar [31], and Rbio software, following the criteria recommended by Cruz et al. [32].

## Results

Table 3 shows the analyses of variance for the agronomic traits, wavelengths, and vegetation indices evaluated in ten soybean cultivars. There were significant differences (p-value ≤ 0.05) between the genotypes (G) for all analyzed variables. The crop season (S) was not significant for the FPH, GNDVI, and SCCCI. It is important to emphasize that all evaluated variables showed a coefficient of variation (CV) below 20%. The wavelengths and VIs showed the lowest CVs, varying from 1.15 (NDVI) to 7.88% (EVI). The CVs of the agronomic traits varied from 9.33 (DM) to 18.89 (SD). These results reveal a high precision of the measurements, especially for the spectral variables, and the possibility of an accurate association between the spectral variables and cycle and yield-related variables.

**Table 3:** P-values for first pod height (FPH), plant height (PH), number of branches (NB), main stem diameter (SD), days to maturity (DM), grain yield (YIE), wavelengths (550, 660, 735 and 790 nm) and vegetation indices (NDVI, SAVI, GNDVI, NDRE, SCCCI, EVI and MSAVI), evaluated in 10 soybean genotypes cultivated in the 2018/2019 and 2019/2020 crop seasons

Variable	Block	Genotypes (G)	Crop season (S)	GxS	Coefficient of variation (%)
FPH	0.12^ns^	0.05*	0.99^ns^	0.05*	13.69
PH	0.09^ns^	0.00*	0.00*	0.00*	12.32
NB	0.24^ns^	0.01*	0.01*	0.01*	18.47
SD	0.31^ns^	0.05*	0.00*	0.18^ns^	18.89
DM	0.41^ns^	0.01*	0.00*	0.00*	9.33
YIE	0.08^ns^	0.00*	0.00*	0.00*	11.64
550	0.44^ns^	0.00*	0.00*	0.00*	2.14
660	0.26^ns^	0.00*	0.01*	0.99^ns^	2.60
735	0.67^ns^	0.00*	0.01*	0.99^ns^	6.07
790	0.18^ns^	0.00*	0.01*	0.99^ns^	6.88
NDVI	0.17^ns^	0.00*	0.00*	0.00*	1.15
SAVI	0.25^ns^	0.00*	0.01*	0.99^ns^	5.82
GNDVI	0.31^ns^	0.00*	0.27^ns^	0.99^ns^	1.43
NDRE	0.42^ns^	0.00*	0.03*	0.99^ns^	2.86
SCCCI	0.17^ns^	0.00*	0.99^ns^	0.99^ns^	3.64
EVI	0.27^ns^	0.00*	0.01*	0.99^ns^	7.88
MSAVI	0.34^ns^	0.00*	0.01*	0.99^ns^	7.30

^ns^ and *not significant and significant at 5% probability by the t-test, respectively

Regarding the grouping of the agronomic trait means (Table 4), the genotype 7739 had the highest FPH, NB, and diameter of the main stem (DS). The genotype 8372 had the highest FPH, NB, and DM, while the genotype Bonus showed the highest PH and SD. The genotype Pop had higher PH, NB, and SD means along with a lower DM. The genotypes Maracai and Foco showed the highest grain yields throughout the crop seasons.

**Table 4:** Grouping of means ± standard deviation for first pod height (FPH), plant height (PH), number of branches (NB), main stem diameter (SD), days to maturity (DM), and grain yield (YIE), evaluated in 10 soybean genotypes cultivated in the 2018/2019 and 2019/2020 crop seasons

Genotypes	FPH (cm)	PH (cm)	NB	SD (mm)	DM	YIE (kg ha^−1^)
7067	13.80b ± 2.61^1^	78.06 ± 7.12b	3.10 ± 0.39b	7.54 ± 0.62b	105.00 ± 2.19f	2905.94 ± 565.64c
7110	12.15 ± 4.79b	67.27 ± 6.76b	3.12 ± 0.52b	6.92 ± 0.81b	100.75 ± 3.04g	2899.01 ± 726.88c
7739	17.18 ± 2.92a	74.20 ± 8.03b	4.56 ± 0.56a	7.75 ± 0.81a	113.63 ± 1.73d	3216.23 ± 317.49b
8372	13.88 ± 4.06b	93.11 ± 7.21a	4.50 ± 0.98a	6.96 ± 0.82b	130.38 ± 1.67a	3313.12 ± 262.27b
Bonus	12.75 ± 2.79b	98.06 ± 5.83a	3.32 ± 0.88b	7.93 ± 0.80a	122.38 ± 1.20b	3266.46 ± 528.76b
Desafio	14.44 ± 3.76a	69.58 ± 5.43b	2.50 ± 0.21b	7.52 ± 0.92b	104.75 ± 1.51f	2806.36 ± 750.63c
Maracai	15.87 ± 6.04a	70.53 ± 6.40b	2.41 ± 0.64b	6.73 ± 0.53b	119.63 ± 0.76c	3834.75 ± 571.64a
Foco	12.05 ± 4.53b	76.58 ± 5.67b	3.05 ± 0.25b	8.07 ± 0.83a	106.38 ± 1.07e	3630.99 ± 592.94a
Pop	13.91 ± 4.97b	91.01 ± 2.89a	4.97 ± 0.61a	7.83 ± 0.68a	101.25 ± 1.46g	3092.82 ± 317.28c
Soyouro	14.59 ± 3.46b	76.32 ± 7.73b	3.35 ± 0.22b	7.00 ± 0.84b	100.63 ± 1.60g	3356.69 ± 426.93b

^1^Means followed by different letters in the same column differ from each other by the Scott–Knott test at 5% probability

The genotype 7739 presented the highest means for all assessed wavelengths, as shown in Table 5. Other genotypes obtained high means for two of the wavelengths, including 8372 (660 and 735 nm), Bonus (550 and 790 nm), Foco, and Maracai (735 and 790 nm). Table 6 shows the mean groupings of the VIs between the genotypes. It is important to note that the genotypes Maracai and Foco belonged to the groups with the highest means for all VIs. The genotypes 7067, 71,110, Bonus, Desafio, Maracai, Foco, and Soyouro obtained the highest means for the GNDVI. For the NDVI, the genotypes 7739, 8372, Bonus, Maracai, and Foco presented the better results.

**Table 5:** Grouping of means ± standard deviation for wavelengths green (550 nm), red (660 nm), near-infrared (735 nm), and infrared (790 nm), evaluated in 10 soybean genotypes cultivated in the 2018/2019 and 2019/2020 crop seasons

Genotypes	550 nm	660 nm	735 nm	790 nm
7067	0.0471 ± 0.0165d^1^	0.0353 ± 0.0344b	0.2761 ± 0.0127b	0.4468 ± 0.0094b
7110	0.0482 ± 0.0370c	0.0350 ± 0.0895b	0.2910 ± 0.0124b	0.4648 ± 0.0073b
7739	0.0540 ± 0.0083a	0.0362 ± 0.0295a	0.3066 ± 0.0118a	0.4848 ± 0.0074a
8372	0.0512 ± 0.0111b	0.0390 ± 0.0877a	0.2855 ± 0.0277a	0.4484 ± 0.0080b
Bonus	0.0537 ± 0.0134a	0.0349 ± 0.0316b	0.3131 ± 0.0071b	0.4989 ± 0.0076a
Desafio	0.0459 ± 0.0309e	0.0334 ± 0.0456b	0.2819 ± 0.0151b	0.4618 ± 0.0111b
Maracai	0.0491 ± 0.0216c	0.0329 ± 0.0356b	0.3029 ± 0.0112a	0.4907 ± 0.0093a
Foco	0.0508 ± 0.0065b	0.0355 ± 0.0435b	0.3030 ± 0.0133a	0.4992 ± 0.0082a
Pop	0.0505 ± 0.0168b	0.0371 ± 0.0428a	0.2818 ± 0.0135b	0.4393 ± 0.0089b
Soyouro	0.0499 ± 0.0239b	0.0355 ± 0.0397b	0.2905 ± 0.0104b	0.4663 ± 0.0074b

^1^Means followed by different letters in the same column differ from each other by the Scott-Knott test at 5% probability

**Table 6:** Grouping of means ± standard deviation for NDVI, SAVI, GNDVI, NDRE, SCCCI, EVI, and MSAVI, evaluated in 10 soybean genotypes in the 2018/2019 and 2019/2020 crop seasons

Genotypes	NDVI	SAVI	GNDVI	NDRE	SCCCI	EVI	MSAVI
7067	0.8593 ± 0.0088b^1^	0.6279 ± 0.0249b	0.8089 ± 0.0494a	0.2360 ± 0.0897a	0.2765 ± 0.0184a	0.3153 ± 0.0204b	0.6754 ± 0.0322b
7110	0.8550 ± 0.0238b	0.6428 ± 0.0422b	0.8109 ± 0.0257a	0.2293 ± 0.0991b	0.2670 ± 0.0710b	0.3266 ± 0.0039b	0.6948 ± 0.0788b
7739	0.8744 ± 0.0044a	0.6585 ± 0.0238a	0.7994 ± 0.0421b	0.2251 ± 0.0494b	0.2614 ± 0.0715b	0.3460 ± 0.0646a	0.7154 ± 0.0242a
8372	0.8675 ± 0.0215a	0.6179 ± 0.0591b	0.7919 ± 0.1158b	0.2210 ± 0.0798b	0.2670 ± 0.0692b	0.3160 ± 0.0779b	0.6626 ± 0.0262b
Bonus	0.8690 ± 0.0056a	0.6724 ± 0.0255a	0.8056 ± 0.0417a	0.2284 ± 0.0994b	0.2633 ± 0.0133b	0.3550 ± 0.0437a	0.7328 ± 0.0254a
Desafio	0.8591 ± 0.0066b	0.6441 ± 0.0321b	0.8181 ± 0.0654a	0.2180 ± 0.0536b	0.2586 ± 0.0043b	0.3249 ± 0.0123b	0.6985 ± 0.0384b
Maracai	0.8688 ± 0.0088a	0.6701 ± 0.0255a	0.8174 ± 0.0502a	0.2361 ± 0.0762a	0.2703 ± 0.0370a	0.3468 ± 0.0325a	0.7325 ± 0.0318a
Foco	0.8784 ± 0.0051a	0.6708 ± 0.0309a	0.8145 ± 0.0594a	0.2441 ± 0.0450a	0.2823 ± 0.0661a	0.3481 ± 0.0136a	0.7304 ± 0.0284a
Pop	0.8558 ± 0.0077b	0.6166 ± 0.0311b	0.7929 ± 0.0600b	0.2413 ± 0.0774a	0.2791 ± 0.0007a	0.3134 ± 0.0508b	0.6590 ± 0.0293b
Soyouro	0.8568 ± 0.0049b	0.6438 ± 0.0293b	0.8063 ± 0.0551a	0.2321 ± 0.0662a	0.2706 ± 0.0011a	0.3299 ± 0.0188b	0.6958 ± 0.0251b

^1^Means followed by different letters in the same column differ from each other by the Scott–Knott test at 5% probability

**Fig. 3:**
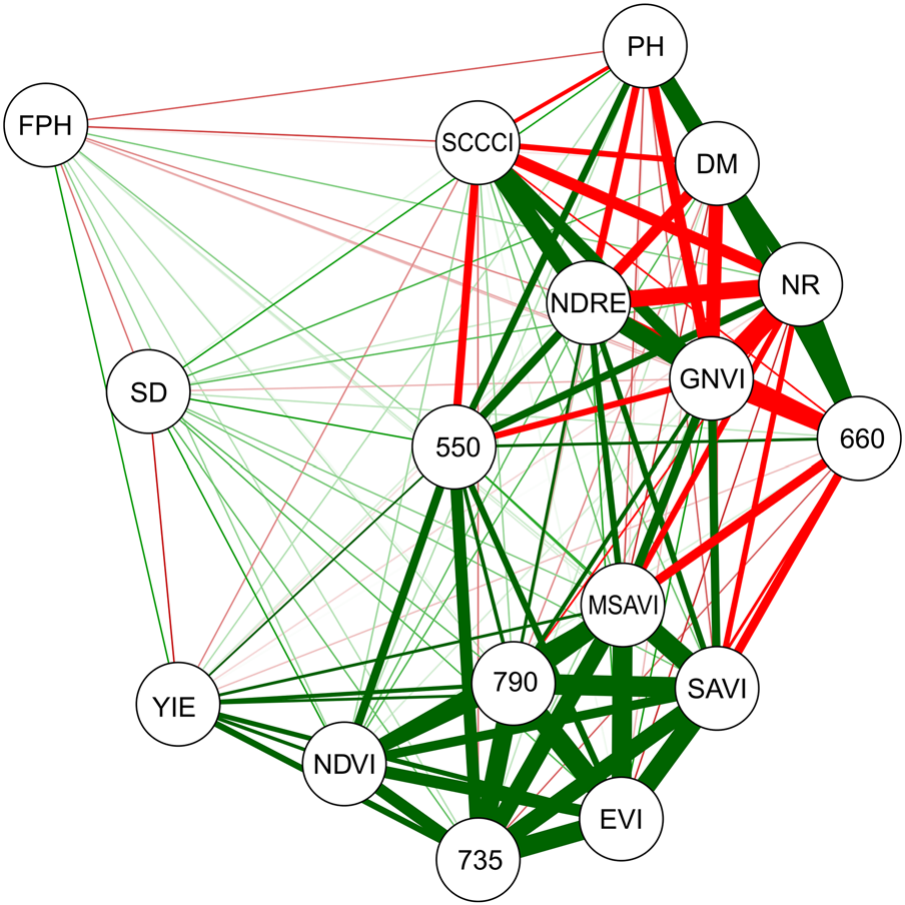
Pearson correlation network between first pod height (FPH), plant height (PH), number of branches (NB), main stem diameter (SD), days to maturity (DM), grain yield (YIE), wavelengths (550, 660, 735 and 790 nm) and vegetation indices (NDVI, SAVI, GNDVI, NDRE, SCCCI, EVI, and MSAVI), evaluated in 10 soybean genotypes in the 2018/2019 and 2019/2020 crop seasons

The Pearson’s correlation network between the evaluated variables is shown in Fig. 3. The YIE was positively correlated with the NDVI and certain wavelengths (735 and 790 nm). The path analysis considering the DM as the principal dependent variable is shown in Table 7. The GNDVI and NDRE vegetation indices had the highest direct effects (module), which were also in the same direction as their correlations with the DM. Table 8 shows the direct and indirect effects of the wavelengths and VIs on the YIE. The NDVI had a higher direct effect (module), which was in the same direction as its correlations with the YIE and MSAVI. The coefficients of determination (R) for the path analysis considering DM as the principal dependent variable (Table 7) was 0.71, while the R of the analysis considering YIE as the principal dependent variable (Table 8) was 0.81. Both R values are considered adequate, revealing that the evaluated variables explained most of the data variation.

**Table 7:** Path analysis for the effects of wavelengths (550, 660, 735 and 790 nm) and vegetation indices (NDVI, SAVI, GNDVI, NDRE, SCCCI, EVI, and MSAVI) on days to maturity (DM), evaluated in 10 soybean genotypes in the 2018/2019 and 2019/2020 crop seasons

Effect	550	660	735	790	NDVI	SAVI	GNDVI	NDRE	SCCCI	EVI	MSAVI
Direct over DM	0.08	0.05	0.09	− 0.01	0.35	− 0.17	− 0.36	− 0.43	0.17	0.07	− 0.11
Indirect via 550	–	0.04	0.06	0.04	0.05	0.03	− 0.04	− 0.04	− 0.05	0.05	0.03
Indirect via 660	0.02	–	− 0.01	− 0.03	0.00	− 0.03	− 0.05	− 0.03	− 0.02	− 0.02	− 0.04
Indirect via 735	0.07	− 0.02	–	0.08	0.07	0.08	0.01	0.01	− 0.01	0.09	0.08
Indirect via 790	− 0.01	0.01	− 0.01	–	− 0.01	− 0.02	− 0.01	− 0.01	0.00	− 0.02	− 0.01
Indirect via NDVI	0.22	0.02	0.26	0.26	–	0.22	0.02	0.08	0.05	0.25	0.22
Indirect via SAVI	− 0.06	0.11	− 0.14	− 0.17	− 0.10	–	− 0.10	− 0.09	− 0.03	− 0.16	− 0.17
Indirect via GNDVI	0.16	0.27	− 0.04	− 0.14	− 0.01	− 0.18	–	− 0.27	− 0.21	− 0.12	− 0.18
Indirect via NDRE	0.21	0.30	− 0.03	− 0.19	− 0.10	− 0.23	− 0.39	–	− 0.40	− 0.14	− 0.23
Indirect via SCCCI	− 0.10	− 0.07	− 0.04	0.03	0.03	0.04	0.13	0.16	–	0.00	0.04
Indirect via EVI	0.04	− 0.03	0.06	0.06	0.05	0.06	0.03	0.02	0.00	–	0.06
Indirect via MSAVI	− 0.03	0.07	− 0.09	− 0.10	− 0.07	− 0.11	− 0.07	− 0.06	− 0.02	− 0.10	–
Total (r)	0.60	0.74	0.09	− 0.17	0.26	− 0.31	− 0.79	− 0.69	− 0.53	− 0.11	− 0.32

Coefficient of determination = 0.71

Effect of the residual variable = 0.35

**Table 8:** Path analysis for the effects of wavelengths (550, 660, 735, and 790 nm) and vegetation indices (NDVI, SAVI, GNDVI, NDRE, SCCCI, EVI, and MSAVI) on grain yield (YIE), evaluated in 10 soybean genotypes in the 2018/2019 and 2019/2020 crop seasons

Effect	550	660	735	790	NDVI	SAVI	GNDVI	NDRE	SCCCI	EVI	MSAVI
Direct over YIE	− 0.27	− 0.16	0.18	0.09	0.34	0.04	− 0.15	− 0.40	0.00	0.07	0.17
Indirect via 550	–	− 0.12	− 0.19	− 0.12	− 0.16	− 0.09	0.15	0.13	0.16	− 0.14	− 0.08
Indirect via 660	− 0.07	–	0.04	0.07	− 0.01	0.10	0.14	0.11	0.06	0.07	0.10
Indirect via 735	0.13	− 0.04	–	0.17	0.13	0.16	0.03	0.01	− 0.04	0.17	0.15
Indirect via 790	0.04	− 0.04	0.08	–	0.06	0.09	0.04	0.04	0.01	0.09	0.08
Indirect via NDVI	0.21	0.02	0.25	0.25	–	0.22	0.02	0.08	0.05	0.25	0.22
Indirect via SAVI	0.01	− 0.02	0.04	0.04	0.02	–	0.02	0.02	0.01	0.04	0.04
Indirect via GNDVI	0.08	0.14	− 0.02	− 0.07	− 0.01	− 0.09	–	− 0.14	− 0.11	− 0.06	− 0.09
Indirect via NDRE	0.20	0.27	− 0.03	− 0.18	− 0.09	− 0.21	− 0.36	–	− 0.36	− 0.14	− 0.22
Indirect via SCCCI	0.00	0.00	0.00	0.00	0.00	0.00	0.00	0.00	–	0.00	0.00
Indirect via EVI	0.04	− 0.03	0.07	0.07	0.05	0.07	0.03	0.02	0.01	–	0.07
Indirect via MSAVI	0.05	− 0.11	0.14	0.16	0.11	0.17	0.10	0.09	0.04	0.16	–
Total (r)	0.41	− 0.09	0.55	0.48	0.46	0.44	0.01	− 0.05	− 0.18	0.50	0.44

Coefficient of determination = 0.81

Effect of the residual variable = 0.33

**Fig. 4:**
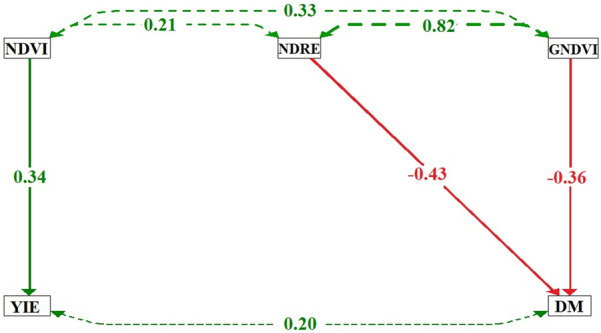
Path diagram for results from Tables 6 and 7 for dependent variables (grain yield—YIE and days to maturity—DM) as function of selected vegetation indices (NDVI, NDRE and GNDVI). Dashed lines indicate Pearson’s correlations between variables, while continuous lines reveal the direct effects obtained by path analysis (previously performed)

The relationships between the NDVI, NDRE, and GNDVI with the grain yield and days to maturity are shown in Fig. 4. The dashed lines between the VIs show a positive correlation and high magnitude between the NDRE and GNDVI. The NDVI presents a direct positive effect on the grain yield. As previously mentioned, there is a direct relationship between both variables, so it is possible to estimate the final production of a crop using NDVI data. The NDRE and GNDVI showed direct negative effects on the days to maturity, meaning that the higher the NDVI and GNDVI, the lower the DM of the crop.

## Discussion

The significance of the other variables can be related to the distinct weather conditions between the crop seasons, such as rainfall and temperature. The interaction (G × S) was significant for the first pod height (FPH), plant height (PH), number of branches (NB), days to maturity (DM), grain yield (YIE), 550 nm wavelength, and NDVI.

The variables differ between the genotypes due to their genetic and morphological characteristics and the environmental conditions to which they were subjected. Overall, the plants showed a low reflectivity in the visible spectrum range (400 to 700 nm) due to the influence of chlorophyll, the most abundant pigment in leaves. The chlorophyll presented a high absorption of blue and red wavelengths, while reflecting the green wavelength, which is remarkable, especially in healthy plants [33].

The presence of genotypes Maracai and Foco in the groups with the highest means for all VIs is interesting, as these genotypes were the ones with the highest grain yield means. This finding supports the existence of a high correlation between the YIE and VIs during the reproductive stage of soybean, in which the plant reaches the maximum leaf area index and consequently has a high photosynthetic rate [34]. Another meaningful relationship was observed for Pop and Soyouro, which were the earliest genotypes and showed the highest means for the NDRE and SCCCI indices. This finding is supported by the association between soybean reflectance and phenological crop stage, in which cultivars with short cycles have faster development and higher chlorophyll concentration [35].

For the VIs, high values were only obtained for the NDVI and GNDVI. This result can be explained by the greater sensitivity of these VIs to the identification of canopy biomass, since both the GNDVI and NDVI are more sensitive to detect differences in the plant canopy [36], especially in terms of chlorophyll content and photosynthetic activity [37]. The VIs EVI, SAVI and MSAVI differ from NDVI and GNDVI especially by using correction factors, such as areas with a high presence of bare soil [6], while NDRE is more sensitive to detecting differences in late stages of growth, characterizing one of the possible reasons why the NDVI and GNVI values are higher.

The positive correlation between YIE x NDVI and wavelengths (735 and 790 nm) indicates that the higher the estimates of these wavelengths and the NDVI, the higher the grain yield achieved by the evaluated genotypes. Such results are relevant because although the grain yield is the most crucial trait in a soybean breeding program, it has low heritability due to the high environmental effect and laborious measurement [16, 38].

In this sense, including the NDVI and 735/790 nm wavelength measurements as auxiliary variables for selecting soybean genotypes is a promising strategy since they are easier to measure, faster to obtain, require less labor, and provide more accurate results compared to grain yield measurements [7, 39]. The NDVI and 735/790 nm wavelengths can remotely measure a large number of candidates for selection [16], which can improve the efficiency of breeding programs. In addition, the NDVI was positively correlated with the 735, 790, and 550 nm wavelengths, which in turn showed a positive correlation with the 660 nm wavelength. There was also a strong negative correlation between DM, GNDVI, and NDRE.

Although important, Pearson’s correlation coefficients can produce misunderstandings regarding the relationship between two variables, which may not be a true cause-and-effect relationship. A high or low correlation coefficient between two variables may result from the effects of a third variable or group of variables, thus not giving the exact relative importance of the direct and indirect effects of these factors [32]. Therefore, we performed path analysis, which investigates cause-and-effect relationships. This analysis promotes a detailed understanding of the effects of the variables involved and justifies the existence of positive and negative correlations (of a high or low magnitude) between the studied variables [40].

However, to obtain the direct and indirect effects by path analysis, the matrix X′X must be well-conditioned. Under the presence of multicollinearity, the variances associated with the path coefficient estimators can reach the highest values, becoming unreliable. Furthermore, the parameter estimates can assume values beyond the parametric space [32]. According to the criteria established in Montgomery and Peck [41], the obtained phenotypic correlation matrix estimates has strong multicollinearity since the condition number (CN) was equal to 521 and 223 when considering the YIE and DM as principal dependent variables, respectively. The CN of the phenotypic correlation matrix is calculated by the ratio of its highest eigenvalue over its lowest eigenvalue. When the condition number is less than 100, multicollinearity is weak; between 100 and 1000, multicollinearity is moderate to strong; finally, when greater than 1000, multicollinearity is severe [41]. Thus, a constant k = 0.05 was added to the X′X diagonal matrix to correct the multicollinearity for both cases.

These results of path analysis on days to maturity reveal a negative cause-and-effect relationship between the VIs and DM. Thus, the higher the values of these indices, the earlier the soybean genotypes are. This is due to the rapid initial development and higher chlorophyll concentration of these genotypes [35].

For path analysis on grain yield, we found a positive cause-and-effect relationship between NDVI and YIE. Thus, the higher the NDVI values, the higher the yield of the soybean genotypes. Lopresti et al. [14] reported that wheat crop monitoring using grain yield maps (obtained using the NDVI) could predict the grain yield 30 days before harvest. The NDVI allows for the monitoring of the soybean biomass growth, which provides information throughout the sub-periods of the crop cycle, thus establishing production estimates [42].

Soybean breeding programs increasingly seek to develop early soybean genotypes to facilitate the cultivation of crops, such as maize and cotton, during the second harvest. Thus, the DM is a continuously evaluated trait in hundreds of genotypes, but there is a lack of information concerning its relationships with the emitted wavelength and vegetation indices. The results provided by the correlation network demonstrate a negative association between the NDRE, GNDVI, and SCCCI with the DM, indicating that genotypes with higher values for these VIs can be selected for earliness. This is an important finding for soybean breeding, as it reveals the possibility of identifying early genotypes by UAV-based HTP using the VIs mentioned. Whereas traditional phenotyping of the soybean cycle is a time and labor-consuming task, requiring daily field visits to count the number of days to maturity, the use of VIs as a tool for selecting early genotypes can contribute to a significant decrease in the time and effort spent on this step of the program.

The acquisition of large-scale phenotypic data has become one of the major bottlenecks hindering crop breeding [22]. Our study provides relevant information to support management and decision-making in soybean breeding since we demonstrate that it is possible to select genotypes for earliness and yield through an easy and economical high-throughput phenotyping approach. Using the approach employed here, which involves the employment of statistical techniques to study the relationship between agronomic traits and VIs as well as the selection of genotypes based on VIs obtained by UAV imagery can increase the efficiency of current breeding programs by enabling large-scale evaluations with time and labor savings. In this sense, further studies addressing yield and maturity prediction of soybean genotypes based on the vegetation indices studied here are very promising and could be used to improve the efficiency of phenotypic evaluations in breeding programs.

## Conclusions

Soybean genotypes with a high grain yield (Maracai and Foco) have higher vegetation index values, especially for the 735 and 790 nm wavelengths and NDVI. This vegetation index has a cause-and-effect relationship with the grain yield of soybean. Our findings suggest that NDVI can be used for high-throughput phenotyping to select genotypes for high grain yield in soybean breeding programs.

The earliest soybean genotypes have a higher NDRE and GNDVI. Due to the requirement for earlier genotypes, the number of days to maturity has been increasingly evaluated in soybean breeding programs. For a cause-and-effect relationship with the DM, we recommend that the NDRE and GNDVI vegetation indices be used for high-throughput phenotyping in soybean breeding programs seeking to select earlier genotypes.

## Data Availability

The datasets used and/or analysed during the current study are available from the corresponding author on reasonable request.
